# Supplementary material for: TRACT revisited: an algebraic solution for determining overall rotational correlation times from cross-correlated relaxation rates
Source: J Biomol NMR. Author manuscript; Available in PMC 2022 Sep 3. (PMC8627365; doi:10.1007/s10858-021-00379-5)
Supplement: 1747980_Sup_info [file NIHMS1747980-supplement-1747980_Sup_info.docx]

**TRACT revisited: an algebraic solution for determining overall rotational correlation times from cross-correlated relaxation rates**

Scott A. Robson^1^, Çağdaş Dağ^1,*^, Hongwei Wu^2^, and Joshua J. Ziarek^1,†^

^1^Indiana University, Department of Molecular and Cellular Biochemistry, 212 S. Hawthorne Drive, Bloomington, IN 47405 USA

^2^Indiana University, Department of Chemistry, 212 S. Hawthorne Drive, Bloomington, IN 47405 USA

*Current address: Koc University, Department of Molecular Biology and Genetics, Nanofabrication and Nanocharacterization Center for Scientific and Technological Advanced Research, and Koc University Isbank Center for Infectious Diseases (KUISCID), Istanbul, 34450 Turkey

^†^Corresponding author: jjziarek@indiana.edu

**Supporting Information**

**Listing S1:** Algebraic Solution for tau_c with and without order parameter S^2^

<https://github.com/nomadiq/TRACT_analysis/blob/master/tract.py>

**import** numpy **as** np


**def** default_tract_params():
 params = {**"h"**: 6.62607004 * (1 / np.power(10, 34, dtype=np.longdouble))}
 params[**"mu_0"**] = 1.25663706 * (
 1 / np.power(10, 6, dtype=np.longdouble)
 ) *# vacuum permeability* params[**"gamma_H"**] = 267.52218744 * np.power(
 10, 6, dtype=np.longdouble
 ) *# proton gyromagnetic ratio* params[**"gamma_N"**] = -27.116 * np.power(
 10, 6, dtype=np.longdouble
 ) *# 15N gyromagnetic ratio* params[**"r"**] = 1.02 * (
 1 / np.power(10, 10, dtype=np.longdouble)
 ) *# internuclear distance* params[**"delta_dN"**] = 160 * (
 1 / np.power(10, 6)
 ) *# diff in axially symetric 15N CS tensor* params[**"theta"**] = 17 * np.pi / 180 *# angle between CSA axis and N-H bond* **return** params


**def** J(w, tc):
 **return** 0.4 * tc / (1 + (w ** 2 * tc ** 2))


**def** J_S2(w, tc, S2):
 **return** S2 * J(w, tc)


*# function that returns evaluation of equation (12) with w_N and constant 'c' above as inputs***def** tc_algebraic(Ra, Rb, field, params=**None**):

 **if not** params: *# use these defaults* params = default_tract_params()

 h = params[**"h"**]
 mu_0 = params[**"mu_0"**]
 gamma_H = params[**"gamma_H"**]
 gamma_N = params[**"gamma_N"**]
 r = params[**"r"**]
 delta_dN = params[**"delta_dN"**]
 theta = params[**"theta"**]

 B_0 = field * np.power(10, 6, dtype=np.longdouble) * 2 * np.pi / gamma_H *# in Tesla* p = (
 mu_0
 * gamma_H
 * gamma_N
 * h
 / (16 * np.pi * np.pi * np.sqrt(2) * np.power(r, 3))
 ) *# DD 1H-15N bond* dN = gamma_N * B_0 * delta_dN / (3 * np.sqrt(2)) *# 15N CSA* w_N = B_0 * gamma_N *# 15N frequency (radians/s)* c = (Rb - Ra) / (2 * dN * p * (3 * np.cos(theta) ** 2 - 1))

 t1 = (5 * c) / 24
 t2 = (336 * (w_N ** 2) - 25 * (c ** 2) * (w_N ** 4)) / (
 24
 * (w_N ** 2)
 * (
 1800 * c * (w_N ** 4)
 + 125 * (c ** 3) * (w_N ** 6)
 + 24
 * np.sqrt(3)
 * np.sqrt(
 21952 * (w_N ** 6)
 - 3025 * (c ** 2) * (w_N ** 8)
 + 625 * (c ** 4) * (w_N ** 10)
 )
 )
 ** (1 / 3)
 )
 t3 = (
 1800 * c * (w_N ** 4)
 + 125 * (c ** 3) * (w_N ** 6)
 + 24
 * np.sqrt(3)
 * np.sqrt(
 21952 * (w_N ** 6)
 - 3025 * (c ** 2) * (w_N ** 8)
 + 625 * (c ** 4) * (w_N ** 10)
 )
 ) ** (1 / 3) / (24 * w_N ** 2)

 **return** t1 - t2 + t3


*# function that returns evaluation of equation (15) with w_N and constant 'c' above as inputs***def** tc_algebraic_S2(Ra, Rb, field, S2, params=**None**):

 **if not** params: *# use these defaults* params = default_tract_params()

 h = params[**"h"**]
 mu_0 = params[**"mu_0"**]
 gamma_H = params[**"gamma_H"**]
 gamma_N = params[**"gamma_N"**]
 r = params[**"r"**]
 delta_dN = params[**"delta_dN"**]
 theta = params[**"theta"**]

 B_0 = field * np.power(10, 6, dtype=np.longdouble) * 2 * np.pi / gamma_H *# in Tesla* p = (
 mu_0
 * gamma_H
 * gamma_N
 * h
 / (16 * np.pi * np.pi * np.sqrt(2) * np.power(r, 3))
 ) *# DD 1H-15N bond* dN = gamma_N * B_0 * delta_dN / (3 * np.sqrt(2)) *# 15N CSA* w_N = B_0 * gamma_N *# 15N frequency (radians/s)* c = (Rb - Ra) / (2 * dN * p * (3 * np.cos(theta) ** 2 - 1))
 w = w_N

 t1 = (
 (
 125 * (c ** 3) * (w ** 6)
 + 24
 * np.sqrt(3)
 * np.sqrt(
 625 * (c ** 4) * (S2 ** 2) * (w ** 10)
 - 3025 * (c ** 2) * (S2 ** 4) * (w ** 8)
 + 21952 * (S2 ** 6) * (w ** 6)
 )
 + 1800 * c * (S2 ** 2) * (w ** 4)
 )
 ** (1 / 3)
 ) / (24 * S2 * w ** 2)

 t2 = (
 -1
 * (336 * (S2 ** 2) * (w ** 2) - 25 * (c ** 2) * (w ** 4))
 / (
 (
 24
 * S2
 * (w ** 2)
 * (
 125 * (c ** 3) * (w ** 6)
 + 24
 * np.sqrt(3)
 * np.sqrt(
 625 * (c ** 4) * (S2 ** 2) * (w ** 10)
 - 3025 * (c ** 2) * (S2 ** 4) * (w ** 8)
 + 21952 * (S2 ** 6) * (w ** 6)
 )
 + 1800 * c * (S2 ** 2) * (w ** 4)
 )
 ** (1 / 3)
 )
 )
 )

 t3 = +(5 * c) / (24 * S2)

 **return** t1 + t2 + t3


*# example*Rb = 64
Ra = 12
field = 750
S2 = 0.86

print(
 **f"\n\u03C4_c = {**tc_algebraic(Ra, Rb, field)*1000000000**:.4f} ns where Rb = {**Rb**} Hz, Ra = {**Ra**} Hz, Field = {**field**} MHz"**)
print(
 **f"\u03C4_c = {**tc_algebraic_S2(Ra, Rb, field, S2)*1000000000**:.4f} ns where Rb = {**Rb**} Hz, Ra = {**Ra**} Hz, Field = {**field**} MHz, S^2 = {**S2**}"**)

print(**"\n\nAdjusting \u03B8 to 20\n"**)
params = default_tract_params()
params[**"theta"**] = 20 * np.pi / 180

print(
 **f"\u03C4_c = {**tc_algebraic(Ra, Rb, field, params)*1000000000**:.4f} ns where Rb = {**Rb**} Hz, Ra = {**Ra**} Hz, Field = {**field**} MHz"**)
print(
 **f"\u03C4_c = {**tc_algebraic_S2(Ra, Rb, field, S2, params)*1000000000**:.4f} ns where Rb = {**Rb**} Hz, Ra = {**Ra**} Hz, Field = {**field**} MHz, S^2 = {**S2**}"**)

**Listing S2:** Numerical Minimization Analysis

<https://github.com/nomadiq/TRACT_analysis/blob/master/tract_minimization_analysis.py>

import numpy as np

from scipy.optimize import minimize_scalar, minimize

# constants

h = 6.62607004 * (1/np.power(10,34, dtype=np.longdouble)) # Plank's

mu_0 = 1.25663706 * (1/np.power(10,6, dtype=np.longdouble)) # vacuum permeability

gamma_H = 267.52218744 * np.power(10,6, dtype=np.longdouble) # proton gyromagnetic ratio

gamma_N = -27.116 * np.power(10,6, dtype=np.longdouble) # 15N gyromagnetic ratio

r = 1.02 * (1/np.power(10,10, dtype=np.longdouble)) # internuclear distance

delta_dN = 160 * (1/np.power(10, 6)) # axially symetric 15N CS tensor

theta = 17*np.pi/180 # angle between CSA axis and NH bond

# field in MHz

field = 750

# derived field value in Tesla

B_0 = field * np.power(10, 6, dtype=np.longdouble) * 2 * np.pi / gamma_H # in Tesla

# equation 7

p = mu_0*gamma_H*gamma_N*h/(16*np.pi*np.pi*np.sqrt(2)*np.power(r,3)) # DD 1H-15N bond

# equation 8

dN = gamma_N*B_0*delta_dN/(3*np.sqrt(2)) # 15N CSA

w_N = B_0 * gamma_N # 15N frequency (radians/s)

# measured relaxation rates

# Panel A Figure 4

Rb = 64 # Hz

Ra = 13 # Hz

#Panel B Figure 4

Rb = 80 # Hz

Ra = 22 # Hz

args = (w_N, Rb, Ra, p, dN, theta)

# Equation 15 function

def objective_function(tau_c, *args):

#spectral density function

def J(w_N, tau_c):

return 0.4*tau_c/(1+(w_N**2*tau_c**2))

#print(*args)

(w_N, Rb, Ra, p, dN, theta) = args # unpack these constants inside the function

return np.abs((4*J(0, tau_c) + 3*J(w_N, tau_c)) - ((Rb - Ra)/(2*p*dN*(3*np.cos(theta)**2-1))))

# guess tau_c

t = 10 * (1/np.power(10,8, dtype=np.longdouble)) # guess 10 ns

res = minimize_scalar(objective_function, args=args, method='Brent')

print(f'Given: field = {field} MHz, Rb = {Rb} Hz and Ra = {Ra} Hz')

print(f'tau_c: {res.x} seconds -- Using Brent Minimization')

print()

res = minimize(objective_function, t, args=args, method='BFGS')

print(f'Given: field = {field} MHz, Rb = {Rb} Hz and Ra = {Ra} Hz')

print(f'tau_c: {res.x} seconds -- Using BFGS Minimization')

print()

res = minimize(objective_function, t, args=args, method='Powell')

print(f'Given: field = {field} MHz, Rb = {Rb} Hz and Ra = {Ra} Hz')

print(f'tau_c: {res.x} seconds -- Using Powell Minimization')

print()

res = minimize(objective_function, t, args=args, method='TNC')

print(f'Given: field = {field} MHz, Rb = {Rb} Hz and Ra = {Ra} Hz')

print(f'tau_c: {res.x} seconds -- Using TNC Minimization')

print()
